# miR2Gene: pattern discovery of single gene, multiple genes, and pathways by enrichment analysis of their microRNA regulators

**DOI:** 10.1186/1752-0509-5-S2-S9

**Published:** 2011-12-14

**Authors:** Chengxiang Qiu, Juan Wang, Qinghua Cui

**Affiliations:** 1 Department of Biomedical Informatics, Peking University Health Science Center, Beijing, 100191, China; 2 MOE Key Lab of Molecular Cardiovascular Science, Peking University, Beijing, 100191, China; 3 Department of Chemical Defense, Institute of Chemical Defense, 1048 Mail Box, Beijing, 102205, China

## Abstract

**Background:**

In recent years, a number of tools have been developed to explore microRNAs (miRNAs) by analyzing their target genes. However, a reverse problem, that is, inferring patterns of protein-coding genes through their miRNA regulators, has not been explored. As various miRNA annotation data become available, exploring gene patterns by analyzing the prior knowledge of their miRNA regulators is becoming more feasible.

**Results:**

In this study, we developed a tool, miR2Gene, for this purpose. Various sets of miRNAs, according to prior rules such as function, associated disease, tissue specificity, family, and cluster, were integrated with miR2Gene. For given genes, miR2Gene evaluates the enrichment of the predicted miRNAs that regulate them in each miRNA set. This tool can be used for single genes, multiple genes, and KEGG pathways. For the KEGG pathway, genes with enriched miRNA sets are highlighted according to various rules. We confirmed the usefulness of miR2Gene through case studies.

**Conclusions:**

miR2Gene represents a novel and useful tool that integrates miRNA knowledge for protein-coding gene analysis. miR2Gene is freely available at http://cmbi.hsc.pku.edu.cn/mir2gene.

## Background

MicroRNAs (miRNAs) are a class of small non-coding RNAs acting as negative gene regulators by binding to the 3'UTR of target mRNAs through base pairing at the post-transcriptional level [1]. Approximately over one third of all genes in the human genome could be regulated by miRNAs [2]. During the past few years, a number of bioinformatics tools have been developed to infer miRNA insights through integrative analysis of miRNAs and their targets [3-7]. These tools help improve our understanding of miRNAs. However, to our knowledge, tools that infer the patterns of protein-coding genes by analyzing the miRNAs that regulate the relevant protein-coding genes are currently unavailable. In recent years, the rapid development of various experiments involving miRNAs has dramatically increased knowledge regarding these regulators. For example, according to the Human microRNA Disease Database (HMDD, http://cmbi.bjmu.edu.cn/hmdd), which manually integrates experimentally supported miRNA-disease associations, the number of reported miRNA-disease associations is quite limited before 2002, but was increased dramatically in recent years, specifically up to 2507 miRNA-disease associations, including 440 distinct miRNA genes and 247 diseases, as stored as of January 2011 [8]. We previously confirmed the usefulness of the prior knowledge for mining novel miRNA patterns for desired miRNAs from biological experiments [9-11]. Meanwhile accumulating knowledge of these regulators makes it possible to explore hidden patterns of protein-coding genes by analyzing the miRNAs that regulate these genes however no such tools are currently available.

For the above purpose, we present a tool, miR2Gene (freely available at http://cmbi.hsc.pku.edu.cn/mir2gene). miR2Gene integrates miRNAs into various miRNA sets according to rules from prior knowledge, such as function, associated disease (HMDD), family, cluster, and tissue specificity. For the given genes, miR2Gene then integrates miRNAs that regulate them and performs enrichment analysis of the predicted miRNA regulators in each predefined miRNA set. The tool then provides the significant miRNA sets, which care the potential patterns of the given genes. Currently, miR2Gene can analyze one single gene, multiple genes, and the KEGG pathways (http://www.genome.jp/kegg/). Finally, we confirmed the usefulness of miR2Gene through case studies.

## Methods

### miR2Gene summary

**Figure 1:**
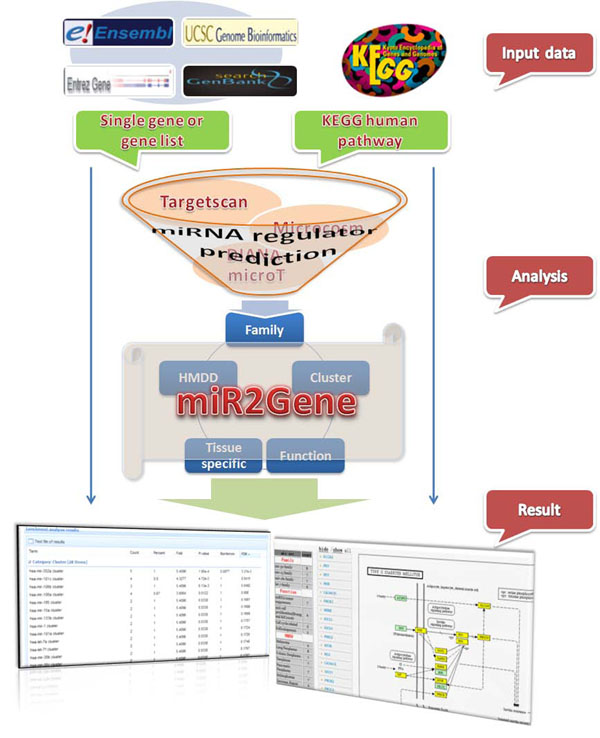
Scheme of the miR2Gene analysis pipeline.

The whole workflow of miR2Gene is shown in Figure 1. For the given protein-coding genes, miR2Gene first predicts the miRNAs that regulates them using different miRNA-target prediction algorithms (TargetScan [2], MicroCosm [12], and DIANA-microT [13]). Then, miR2Gene evaluates the enrichment of the predicted miRNA regulators of the given genes in the predefined miRNA sets. After submitting a task, the results are shown in a new page. For different tasks (single genes, multiple genes, and KEGG pathways), exact procedures have some differences. A tutorial page is provided to make miR2Gene user-friendly. For each specific task, a summarized analysis wizard is also provided in the specific analysis page.

### Input data

When a specific task, such as analysis of one single gene, multiple genes, or one KEGG pathway is selected, the user needs to enter specific input data for the specific task. For single or multiple genes, the user needs to first input the gene name or ID for the specific gene identifiers. Currently, miR2Gene supports seven types of gene identifiers, such as the Official Gene Symbol, Entrez Gene ID, Ensembl Gene ID, Ensembl Transcript ID, UCSC gene ID, Refseq mRNA ID, and GeneBank Accession Number. For multiple genes, they should be arranged in one column and each row represents only one gene. We provide one parameter “set the threshold value” for the analysis of multiple genes. The threshold means that only the miRNAs that regulate no less than the “threshold” of given genes are considered in later analysis. For both single gene analysis and multiple gene analysis, the user can view the predicted miRNA regulators in the corresponding analysis pages. For the KEGG pathway analysis, the user needs to select the desired KEGG pathway first, and then determine whether to analyze the pathway genes individually or analyze them as a whole. The next procedure for all three types of tasks is selecting a method to predict the miRNAs that regulate the given protein-coding genes. miR2Gene provides three choices, namely, TargetScan [2], MicroCosm[12], and DIANA-microT [13] for predicting the miRNA regulators. We downloaded the TargetScan predictions (version 5.1) from http://www.targetscan.org/, the MicroCosm predictions (version 5) from http://www.ebi.ac.uk/enright-srv/microcosm/htdocs/targets/v5/, and the DIANA-microT predictions (version 3.0) from http://diana.cslab.ece.ntua.gr/microT.

### Enrichment analysis of predefined miRNA sets to the predicted regulatory miRNAs for given genes

We used the hypergeometric test to determine the significant enrichment of each miRNA set for the predicted regulatory miRNAs for the given genes, as we previously described [9]. The hypergeometric test generates the significance (P-value) and calculates the fold of enrichment for each miRNA set. The fold value is calculated by dividing the actual with the expected number of predicted miRNAs matched in corresponding miRNA set. The percentage of matched miRNAs in the corresponding miRNA set is also given. Considering that miR2Gene analyzes multiple miRNA sets for the same input dataset, two methods for multiple comparison correction, Bonferroni and FDR, are provided to correct the original P-values.

### Outputs

The result of the desired task is shown in a new page. For analyzing single genes or multiple genes, the miRNA sets that have at least one match in the predicted miRNAs are shown. The miRNA sets are arranged in five categories, namely Cluster, Family, Function, HMDD (miRNA-associated diseases), and TissueSpecific (miRNA tissue specificity, which was obtained from the study of Lu et al. [8]). The miRNA functional set were manually curated from literature. We obtained the miRNA family set and miRNA cluster set from the miRBase database [14]. The user can rank the results by Count (number of matched miRNAs), Percent (percentage of matched miRNAs in corresponding miRNA set), Fold (the actual matched number/expected matched number), P-value, Bonferroni (Bonferroni-corrected P-value), and FDR (FDR-corrected P-value). The significantly enriched miRNA sets are considered as putatively associated with the given protein-coding gene(s). One important point that the user should remember is that the discovered pattern in the Function category could be sometimes reversed because of the inverse regulatory relationship between the given genes and their miRNA regulators.

For the analysis of the KEGG pathways, the result page shows three panels. The left panel lists the significant enriched (FDR≤0.05) miRNA sets arranged in different categories and ranked by significance. The middle panel lists all genes included in the input KEGG pathway. The right panel is the figure presentation of the pathway. Clicking any miRNA set in the left panel with highlight the corresponding genes significantly regulated by the miRNA set in the pathway figure at the right panel. The two buttons in the middle panel can show (highlight) or hide all genes that have at least one significantly enriched miRNA set. Clicking any gene listed in the middle panel will list all significantly enriched miRNA sets associated with the selected gene. The miR2Gene provides links to KEGG.

## Results

To confirm the usefulness of miR2Gene in gene pattern discovery, we chose the gene “ABL2” and the KEGG pathway “cell cycle” as examples for tasks of single gene and pathway analyses. Analysis of multiple genes is similar with that of single gene analysis.

For analysis of ABL2, miR2Gene found that the predicted miRNAs (obtained by TargetScan) that regulate ABL2 are significantly enriched in Cluster mir-302a (FDR = 3.37×10^-3^), mir-181c (FDR = 0.04), and mir-106b (FDR = 0.05), Family let-7, mir-30, mir-17, mir-15, mir-181, mir-302, mir-148, and mir-25. Among these miRNA sets, some of them are well known to be associated with cancer, i.e. let-7 family and mir-17 cluster [8,10]. For the Function category (Additional File 1), the significant miRNA sets include Function miRNA tumor suppressors (FDR = 5.87×10^-8^), anti-cell proliferation (FDR = 2.74×10^-7^), human embryonic stem cell (FDR = 1.29×10^-6^), hormones regulation (FDR = 2.63×10^-4^), cell cycle (FDR = 2.64×10^-4^), folliculogenesis (FDR = 3.51×10^-3^), onco-miRNAs (FDR = 5.28×10^-3^), granulopoiesis (FDR = 6.36×10^-3^), immune response (FDR = 7.80×10^-3^), bone regeneration (FDR = 0.01), apoptosis (FDR = 0.03) and cell proliferation (FDR = 0.05). The analysis in the HMDD category showed that 76% (26/34) of the significant diseases is cancer (Additional File 1). These results indicate that ABL2 is strongly related with cancer. Furthermore, the miRNA sets “miRNA tumor suppressors” is among the top significant sets. Because miRNAs mainly negatively regulate target genes, the above result suggests that ABL2 may act mainly as an oncogene. Indeed, according to the annotation of NCBI (http://www.ncbi.nlm.nih.gov/), ABL2 is a member of the Abelson family of nonreceptor tyrosine protein kinase genes and is v-abl Abelson murine leukemia viral oncogene homolog 2. Interestingly, almost all of the currently reported ABL2-associated cancers have been identified successfully through miR2Gene analysis, including melanoma [15] (FDR = 9.13×10^-9^, rank No.1 in all diseases by miR2Gene), lymphoma [16] (FDR = 4.23×10^-3^) and leukemia [17,18] (FDR = 1.10×10^-3^). Analysis also showed that ABL2 is strongly associated with digestive system cancer (FDR = 3.54×10^-5^), which is further supported by two studies that found ABL2 is involved in gastrointestinal stromal tumors (GISTs) [19,20]. miR2Gene did not directly identify GISTs because GISTs-associated miRNAs are not presently reported. Therefore, these data are not integrated with miR2Gene. Overall, the results show a high accuracy of miR2Gene prediction, suggesting that miR2Gene is a useful tool for gene pattern discovery. Non-cancer diseases showing strong significance through miR2Gene analysis include heart failure (FDR = 4.91×10^-8^, rank No. 4), Schizophrenia (FDR = 2.07×10^-4^), and autistic disorder (FDR = 5.47×10^-3^). Although no study provides evidence for the associations of these disease and ABL2, ABL2 may be a potential molecule associated with these diseases. Interestingly, ABL2 has a role in the KEGG ErbB signaling and viral myocarditis pathways, both of which are associated with heart function, suggesting that ABL2 has a role in heart function and could therefore be associated with heart failure. For the predicted functions, most of them, except for cancer-associated functions do not have direct evidences although several have some indications. For example, the function "granulopoiesis" could be supported indirectly by its well-known involvement in leukemia.

**Figure 2:**
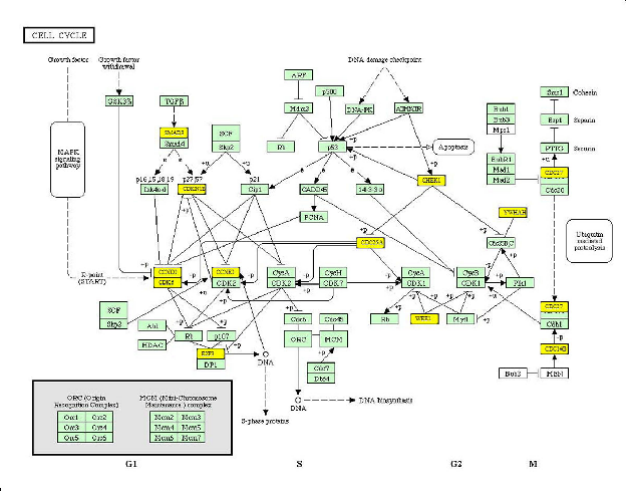
The cell cycle pathway and the significant genes on cell cycle-related miRNAs. The genes whose miRNA regulators are significantly enriched in the cell cycle-related miRNA set are highlighted in yellow.

For the cell cycle pathway analysis, miR2Gene predicted that the mir-302 cluster is the most significant miRNA cluster and the mir-15 family is the most significant miRNA family. Indeed, mir-302 cluster was actually confirmed to be induced by Oct4/Sox2 and it regulates multiple cell cycle regulators. Inhibition of mir-302 causes human embryonic stem cells to accumulate in the G1 phase [21]. The mir-15 family, also known as the mir-16 family, was also confirmed to induce cell cycle arrest by regulating several cell cycle genes [22]. Various types of cancers occupy the top significant locations of the HMDD category, suggesting that cell cycle pathway is strongly related with cancer. The only non-cancer disease among the top locations is heart failure. Moreover, the heart-specific miRNA set is shown as the most significant set in the TissueSpecific category. These results suggest that heart function is also strongly associated with the cell cycle. The “cell cycle” miRNA set is one of the most significant sets in the Function category (rank No. 2). Figure 2 shows more details regarding the cell cycle-related miRNAs involved in the regulation of the cell cycle pathway. The miR2Gene shows that multiple genes in the cell cycle pathway are significantly preferred to be regulated by the cell cycle-related miRNA set. This result was confirmed by Carleton et al., who noted that some genes in the cell cycle pathway such as cyclin protein, CDK6/4, CDK2, E2F, CDC, WEE1 and CHEK1 are miRNA targets and their interactions are involved in cell cycle regulation [23]. miR2Gene also shows that miRNAs seem to take part more in the G1 phase (Figure 2). Although the miR2Gene prediction result on the cell cycle pathway needs further experimental confirmation and support, the new patterns provide new insights into the cell cycle through miRNAs.

## Discussion

By enrichment analysis of miRNAs that regulate the given gene, miR2Gene is able to mine patterns of the given protein-coding genes. Therefore, miR2Gene represents a novel tool in this topic. The results showed that this tool is useful. However, limitations exist in this tool. The major limitation is that currently the data of miRNA set is limited, which may result to bias in the analysis. Another limitation is that the prediction of miRNA-target pairs has high false positives and high false negatives. This also may produce bias in the analysis. We believe that as more miRNA sets are collected and more accurate miRNA-target prediction tools becomes available, miR2Gene would produce more reliable result.

## Conclusions

In recent years, tools have been developed to infer biological insights of miRNAs through integrative analysis of miRNAs and their targets. However, tools for the reverse problem, that is, inferring the biological insights of protein-coding genes through their miRNA regulators are not available because of the limited prior knowledge regarding miRNAs. Considering that a majority of protein-coding genes are putative targets of miRNAs, exploring novel patterns of protein-coding genes through integrative analysis of the miRNAs that regulate them has become increasingly interesting. As prior knowledge regarding miRNAs is accumulating rapidly, developing tools for the above purpose is becoming more feasible. In this study, we developed a tool, miR2Gene, to address this problem. For given protein-coding genes, miR2Gene first predicts the miRNAs that regulate the input genes and then performs enrichment analysis of the predefined miRNA knowledge in the predicted miRNAs. miR2Gene supports three types of analysis, namely single genes, multiple genes, and KEGG pathways. Moreover, the usefulness of miR2Gene has been confirmed through two case studies. Currently, miR2Gene is only used for human genes and pathways, but can easily be extended to other species when sufficient miRNA prior knowledge becomes available.

## Competing interests

The authors declared that they have no competing interests.

## Authors’ contributions

QC designed the study and wrote the manuscript. CQ implemented the algorithms and built the web-server. JW performed the analysis.

## Supplementary Material

Additional File 1 miRNA sets that are significantly enriched in the miRNAs that are predicted to regulate ABL2 and their statistical results. Click here for file
