# Supplementary material for: miR2Gene: pattern discovery of single gene, multiple genes, and pathways by enrichment analysis of their microRNA regulators
Source: BMC Syst Biol. 2011 Dec 14;5(Suppl 2):S9. doi: 10.1186/1752-0509-5-S2-S9 (PMC3287489; doi:10.1186/1752-0509-5-S2-S9)
Supplement: Additional File 1 — miRNA sets that are significantly enriched in the miRNAs that are predicted to regulate ABL2 and their statistical results. [file 1752-0509-5-S2-S9-S1.doc]

Additional File 1. miRNA sets that are significantly enriched in the miRNAs that are predicted to regulate ABL2 and their statistical results.

|  | miRNA set | Count | Percent | Fold | P-value | Bonferroni | FDR |
| --- | --- | --- | --- | --- | --- | --- | --- |
| Cluster | mir-302a | 5 | 1 | 5.41 | 1.95E-04 | 0.0877 | 3.37E-03 |
|  | mir-181c | 4 | 0.8 | 4.33 | 4.72E-03 | 1 | 0.0415 |
|  | mir-106b | 3 | 1 | 5.41 | 6.13E-03 | 1 | 0.0492 |
| Family | let-7 | 9 | 1 | 5.41 | 1.70E-07 | 7.83E-05 | 6.52E-06 |
|  | mir-30 | 5 | 1 | 5.41 | 0.00019524 | 0.0877 | 3.40E-03 |
|  | mir-17 | 6 | 0.75 | 4.06 | 0.00069573 | 0.3124 | 9.19E-03 |
|  | mir-15 | 4 | 1 | 5.41 | 0.00109977 | 0.4938 | 0.0128 |
|  | mir-181 | 4 | 1 | 5.41 | 0.00109977 | 0.4938 | 0.013 |
|  | mir-302 | 4 | 0.8 | 4.33 | 0.00471787 | 1 | 0.0432 |
|  | mir-148 | 3 | 1 | 5.41 | 0.00613119 | 1 | 0.0501 |
|  | mir-25 | 3 | 1 | 5.41 | 0.00613119 | 1 | 0.051 |
| Function | miRNA tumor suppressors | 23 | 0.62 | 3.36 | 0 | 2.93E-07 | 5.87E-08 |
|  | anti-cell proliferation(Hwang etal BJC2006) | 11 | 1 | 5.41 | 0 | 2.19E-06 | 2.74E-07 |
|  | Human embryonic stem cell (hESC) regulation | 35 | 0.41 | 2.23 | 3.00E-08 | 1.29E-05 | 1.29E-06 |
|  | Hormones regulation | 25 | 0.40 | 2.18 | 0.0000106 | 4.76E-03 | 2.63E-04 |
|  | Cell cycle related | 26 | 0.39 | 2.13 | 0.00001114 | 5.00E-03 | 2.64E-04 |
|  | Folliculogenesis | 6 | 0.86 | 4.64 | 0.00020432 | 0.0917 | 3.51E-03 |
|  | onco-miRNAs | 14 | 0.45 | 2.44 | 0.00035311 | 0.1585 | 5.28E-03 |
|  | Granulopoiesis | 7 | 0.7 | 3.79 | 0.00043885 | 0.197 | 6.36E-03 |
|  | Immune response | 18 | 0.38 | 2.07 | 0.00057314 | 0.2573 | 7.80E-03 |
|  | Bone regeneration | 14 | 0.41 | 2.23 | 0.00113355 | 0.509 | 0.0133 |
|  | immune system(Xiao's Cell2009) | 9 | 0.5 | 2.70 | 0.00193409 | 0.8684 | 0.0212 |
|  | Apoptosis | 16 | 0.36 | 1.97 | 0.00240194 | 1 | 0.0251 |
|  | Cell proliferation | 11 | 0.39 | 2.13 | 0.00641976 | 1 | 0.0518 |
| HMDD | Melanoma | 29 | 0.58 | 3.14 | 0 | 9.13E-09 | 9.13E-09 |
|  | Ovarian Neoplasms | 31 | 0.51 | 2.79 | 0 | 9.31E-08 | 3.87E-08 |
|  | Neoplasms | 28 | 0.55 | 2.97 | 0 | 1.47E-07 | 4.66E-08 |
|  | Heart Failure | 42 | 0.41 | 2.23 | 0 | 1.55E-07 | 4.91E-08 |
|  | Breast Neoplasms | 35 | 0.45 | 2.43 | 0 | 6.65E-07 | 1.11E-07 |
|  | Pituitary Neoplasms | 11 | 1 | 5.41 | 0 | 2.19E-06 | 3.13E-07 |
|  | Lung Neoplasms | 32 | 0.44 | 2.40 | 2.00E-08 | 6.82E-06 | 7.58E-07 |
|  | Pancreatic Neoplasms | 21 | 0.57 | 3.07 | 5.00E-08 | 2.09E-05 | 1.90E-06 |
|  | Digestive System Neoplasms | 8 | 1 | 5.41 | 0.00000103 | 4.60E-04 | 3.54E-05 |
|  | Head and Neck Neoplasms | 16 | 0.57 | 3.09 | 0.00000235 | 1.06E-03 | 7.54E-05 |
|  | Lymphoma, Primary Effusion | 9 | 0.82 | 4.43 | 0.00000691 | 3.10E-03 | 1.91E-04 |
|  | Sarcoma, Kaposi | 9 | 0.82 | 4.43 | 0.00000691 | 3.10E-03 | 1.94E-04 |
|  | Schizophrenia | 13 | 0.62 | 3.35 | 0.00000724 | 3.25E-03 | 2.07E-04 |
|  | Prostatic Neoplasms | 17 | 0.5 | 2.70 | 0.00001278 | 5.74E-03 | 2.87E-04 |
|  | Carcinoma, Non-Small-Cell Lung | 8 | 0.8 | 4.33 | 0.00003324 | 0.0149 | 7.11E-04 |
|  | Colonic Neoplasms | 17 | 0.46 | 2.49 | 0.00005382 | 0.0242 | 1.06E-03 |
|  | Leukemia, Lymphocytic, Chronic, B-Cell | 12 | 0.57 | 3.09 | 0.00005424 | 0.0244 | 1.10E-03 |
|  | Lymphoma | 8 | 0.67 | 3.61 | 0.0002635 | 0.1183 | 4.23E-03 |
|  | Autistic Disorder | 14 | 0.45 | 2.44 | 0.00035311 | 0.1585 | 5.47E-03 |
|  | Adrenocortical Carcinoma | 18 | 0.38 | 2.07 | 0.00057314 | 0.2573 | 8.04E-03 |
|  | Stomach Neoplasms | 15 | 0.41 | 2.19 | 0.0008923 | 0.4006 | 0.0114 |
|  | Leukemia, B-Cell | 7 | 0.64 | 3.44 | 0.00102578 | 0.4606 | 0.0127 |
|  | Skin Neoplasms | 4 | 1 | 5.41 | 0.00109977 | 0.4938 | 0.0127 |
|  | Hepatitis C | 7 | 0.58 | 3.16 | 0.00209298 | 0.9397 | 0.0224 |
|  | Endometriosis | 10 | 0.45 | 2.46 | 0.00264017 | 1 | 0.0269 |
|  | Carcinoma, Hepatocellular | 13 | 0.39 | 2.13 | 0.00285297 | 1 | 0.0285 |
|  | Alzheimer Disease | 5 | 0.71 | 3.86 | 0.00298899 | 1 | 0.0286 |
|  | Hepatitis B | 5 | 0.71 | 3.86 | 0.00298899 | 1 | 0.0292 |
|  | Azoospermia | 7 | 0.54 | 2.91 | 0.00385618 | 1 | 0.0361 |
|  | Glioma | 4 | 0.8 | 4.33 | 0.00471787 | 1 | 0.0424 |
|  | Lymphoma, B-Cell | 8 | 0.47 | 2.55 | 0.00571625 | 1 | 0.0483 |
|  | Musculoskeletal Abnormalities | 3 | 1 | 5.41 | 0.00613119 | 1 | 0.0494 |
|  | Asthma | 3 | 1 | 5.41 | 0.00613119 | 1 | 0.0497 |
|  | ACTH-Secreting Pituitary Adenoma | 5 | 0.63 | 3.38 | 0.00681109 | 1 | 0.0519 |
| TissueSpecific | Heart&Muscle | 7 | 0.78 | 4.21 | 0.00015492 | 0.0696 | 2.90E-03 |
